# P-1113. Treatment Patterns and Clinical Outcomes in Hospitalized Patients with Febrile Neutropenia treated with Ceftolozane/Tazobactam: A Subgroup Analysis of the SPECTRA study

**DOI:** 10.1093/ofid/ofae631.1300

**Published:** 2025-01-29

**Authors:** Yanbing Zhou, Alex Soriano, David Paterson, Florian Thalhammer, Stefan Kluge, Pierluigi Viale, Mike Allen, Brune Akrich, Emre Yucel, Huina Yang, Sundeep Kaul

**Affiliations:** Merck, Rahway, New Jersey; Hospital Clínic de Barcelona, Barcelona, Catalonia, Spain; National University of Singapore, Singapore; Medizinische Universität Wien, Vienna, Wien, Austria; Department of Intensive Care, University Medical Center Hamburg-Eppendorf, Hamburg, Hamburg, Germany; Infectious Diseases Unit, Department of Medical and Surgical Sciences, Policlinico Sant'Orsola Malpighi, University of Bologna, Bologna, Italy, Bologna, Emilia-Romagna, Italy; MSD, UK, Ltd., London, England, United Kingdom; Merck Research Labs, MSD, Puteaux, Ile-de-France, France; Merck & Co., Inc., North Wales, Pennsylvania; Tan Tock Seng Hospital, Singapore, Not Applicable, Singapore; Harefield hospital, london, England, United Kingdom

## Abstract

**Background:**

This sub-group analysis of the Study of Prescribing Patterns and Effectiveness of Ceftolozane/Tazobactam(C/T) Real-world Analysis (SPECTRA) described treatment patterns and outcomes in hospitalized patients with Febrile Neutropenia (FN).

**Table 1.**
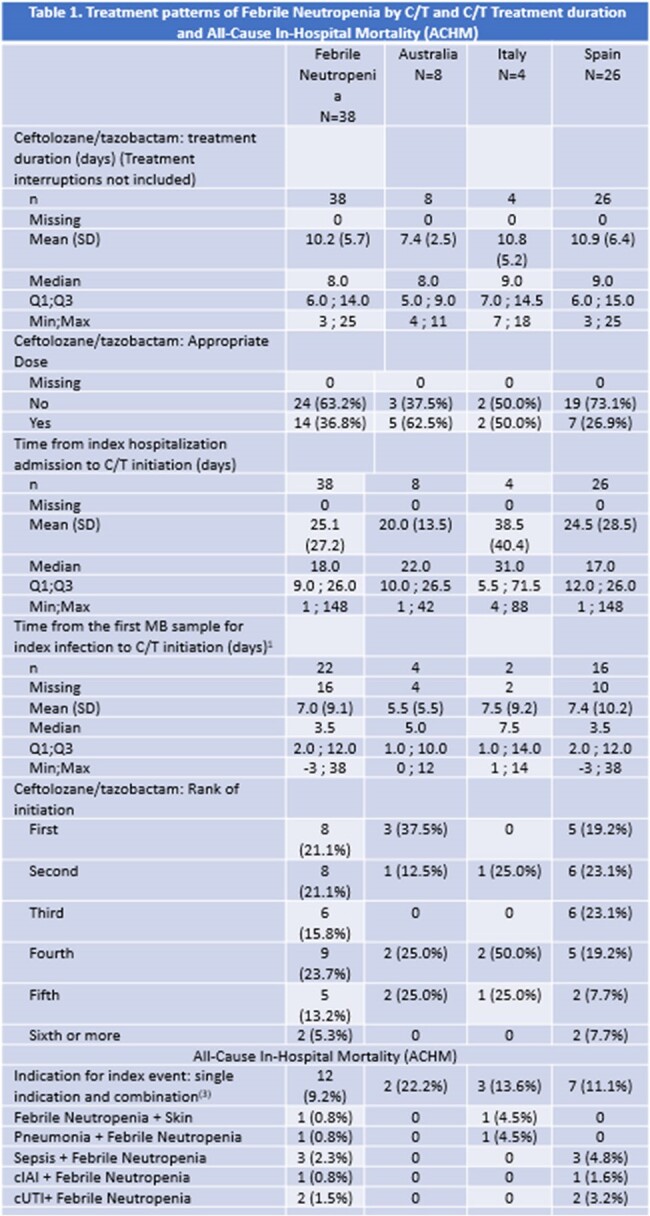
Treatment patterns of Febrile Neutropenia by C/T and C/T Treatment duration and All-Cause In-Hospital Mortality (ACHM) Abbreviations: CRI= Chronic Respiratory Infection cIAI= Complicated Intra-abdominal Infection cUTI= Complicated Urinary Tract Infection

(1) For patients aged 90 or older the HCP was asked not to enter the exact age and to check a specific box. Therefore, patients aged 90 or older are included in 'Missing' and are not taken into account in the calculation of mean, median, etc.

(2) Several indications may have been reported for the same patient.

(3) A patient is counted once for each single indication or combination of indications.

**Methods:**

SPECTRA was a multicenter, observational study, in hospitalized adult patients treated with C/T for ≥48 hours in 7 countries (n=617). Medical records were extracted, covering 6 months prior to the index date, up to 30 days after the last dose of C/T or until death. Descriptive statistics were reported here on treatment patterns, all-cause in-hospital mortality (ACHM), clinical success and microbiologic response in cases of Febrile Neutropenia (FN, n=38).

**Table 2.**
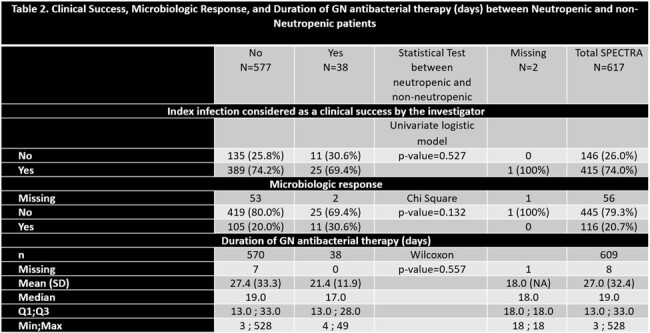
Clinical Success, Microbiologic Response, and Duration of GN antibacterial therapy (days) between Neutropenic and non-Neutropenic patients 1Analysis includes only those with known status for clinical success, and only those with known status for microbiological response. Responses of 'Unknown' are considered as missing data. Microbiological response was defined as negative culture result at site of index infection with same organism post C/T administration.

**Results:**

The median duration of C/T treatment was 8 days, median time from index hospitalization admission to C/T initiation was 18 days, and from the first microbiological sample to C/T initiation 3.5 days (Table 1). ACHM was 9.2% (n=12). Clinical success was 69.4% (n=25) (Table 2). 63% of the patients (n=24) received suboptimal doses for treatment. MDR Pseudomonas aeruginosa and, ESBL-producing Enterobacterales were isolated in 15 and 4 patients, respectively. One had a clinical diagnosis of both pneumonia and sepsis, 2 patients with pneumonia, 4 with respiratory related infections, 1 with skin infection, 3 with sepsis, 1 with cIAI, and 2 with cUTI, in addition to FN. Microbiologic response was achieved for 30.6% of the patients, who received a median of 17 days of GN antibacterial therapy.

**Table 3:**
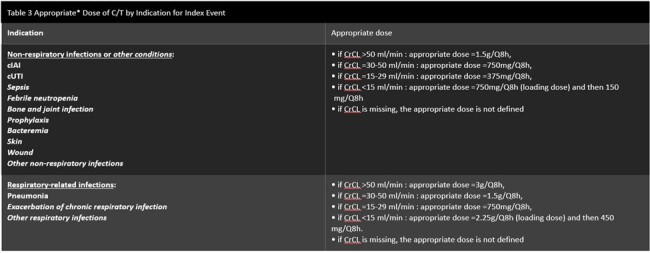
Appropriate* Dose of C/T by Indication for Index Event *If the dose received was different (lower or higher) from the appropriate dose as defined, then the patient was considered as not having received the appropriate dose. If the dose received was different (lower or higher) from the appropriate dose on the first day but was then adjusted to the appropriate dose, then the patient was considered as having received the appropriate dose. For patients having both respiratory and non-respiratory infection, the appropriate dose for respiratory infection (=the highest dose) was considered as the appropriate dose for the patient.

**Conclusion:**

These findings demonstrate potential new findings for the treatment of febrile neutropenia. However, further research is needed to explore its full potential and optimize dosing strategies for improved outcomes.

**Disclosures:**

**Yanbing Zhou, PhD**, Merck: I am a full time Merck Employee and own stocks in the retirement plan provided by Merck.|Merck: Stocks/Bonds (Public Company) **David Paterson**, bioMerieux: Grant/Research Support|bioMerieux: Honoraria|Merck: Advisor/Consultant|Merck: Grant/Research Support|Merck: Honoraria|Pfizer: Advisor/Consultant|Pfizer: Grant/Research Support|Pfizer: Honoraria|Shionogi: Grant/Research Support|Shionogi: Honoraria **Florian Thalhammer, MD**, MSD: Advisor/Consultant **Stefan Kluge, Prof. Dr. med.**, Merck & Co: Advisor/Consultant|Merck & Co: Board Member **Mike Allen, PhD**, Merck: I am a full time Merck Employee and own stocks in the retirement plan provided by Merck.|Merck: Stocks/Bonds (Public Company) **Brune Akrich, MD**, Merck: I am a full time Merck Employee and own stocks in the retirement plan provided by Merck.|Merck: Stocks/Bonds (Public Company) **Emre Yucel, PhD**, Merck: I am a full time Merck Employee and own stocks in the retirement plan provided by Merck.|Merck: Stocks/Bonds (Public Company)

